# Methylation-Mediated Silencing of miR-124-3 Regulates LRRC1 Expression and Promotes Oral Cancer Progression

**DOI:** 10.3390/cancers17071136

**Published:** 2025-03-28

**Authors:** Shin-Wei Liao, Xiao-Hui Liao, Shao-Huang Wu, Yu-Fen Li, Pin-Yi Chen, Yi-Ling Wang, Yin-Che Lu, Chien-Kuo Tai

**Affiliations:** 1 Department of Biomedical Sciences, National Chung Cheng University, Chia-Yi 621, Taiwan; ssaic2000@gmail.com (S.-W.L.);; 2 Department of Public Health, China Medical University, Taichung 404, Taiwan; 3 Department of Nursing, Min-Hwei Junior College of Health Care Management, Tainan 736, Taiwan; 4 Division of Hematology-Oncology, Ditmanson Medical Foundation Chia-Yi Christian Hospital, Chia-Yi 600, Taiwan

**Keywords:** DNA methylation, miR-124-3, LRRC1, oral cancer, tumor suppressor

## Abstract

**Background:** Epigenetic alterations, including DNA methylation, play a crucial role in the development of oral squamous cell carcinoma (OSCC) by regulating the expression of tumor suppressor genes and oncogenes. This study investigated the methylation status of miR-124-3 and its role in OSCC progression. **Methods:** This study applied the Illumina Infinium MethylationEPIC BeadChip assay to profile >850,000 CpG sites in paired OSCC and normal tissues. The methylation data were validated by further analyzing the methylation level of miR-124-3 by using a bisulfite pyrosequencing assay. We investigated whether miR-124-3 acts as a tumor suppressor by establishing miR-124-3-overexpressing OSCC cells and subjecting them to cell proliferation, colony formation, and migration assays. Dual-luciferase reporter assay was used to validate the target genes of miR-124-3 in OSCC cells. **Results:** The Infinium MethylationEPIC BeadChip and bisulfite pyrosequencing assays consistently identified hypermethylation of miR-124-3 in OSCC tissues relative to normal oral tissues. It was especially notable that miR-124-3 methylation levels were markedly higher in late-stage tumors than in early-stage, and differed significantly between early-stage tumor and normal tissues, indicating that miR-124-3 methylation is an early event in OSCC development. Methylation of miR-124-3 contributes markedly to the downregulation of the gene, leading to the increased expression of its target gene, leucine-rich repeat-containing 1 (LRRC1), which is considered to be positively associated with cancer progression. Moreover, overexpression of miR-124-3 suppressed the proliferation and migration of OSCC cells, while silencing the expression of LRRC1 produced similar tumor-suppressive effects. Luciferase reporter assays confirmed that miR-124-3 directly targets the 3′ untranslated region of LRRC1 to downregulate LRRC1 expression. **Conclusions:** Hypermethylation-mediated downregulation of miR-124-3 results in increased LRRC1 expression, which drives OSCC progression. These findings highlight DNA methylation of miR-124-3 as a potential biomarker for the early detection of OSCC and a therapeutic target for OSCC treatments.

## Simple Summary

Abnormal DNA methylation in the promoter of tumor suppressor genes and dysregulated microRNA expression are detected early in the formation of tumor cells and have been shown to influence tumor malignancy. This study investigated the methylation status of miR-124-3 and its role in oral squamous cell carcinoma (OSCC) progression. The Infinium MethylationEPIC BeadChip and bisulfite pyrosequencing assays consistently identified hypermethylation of miR-124-3 in OSCC tissues relative to normal oral tissues. Methylation of miR-124-3 contributes markedly to the downregulation of the gene, leading to the increased expression of its target gene, leucine-rich repeat-containing 1, which is considered to be positively associated with cancer progression. These findings highlight DNA methylation of miR-124-3 as a potential diagnostic biomarker for the early detection of OSCC and a therapeutic target for OSCC treatments.

## 1. Introduction

Head and neck squamous cell carcinoma (HNSCC) is the sixth-most-prevalent cancer globally, with an estimated 900,000 new cases being diagnosed and over 400,000 deaths attributed to the disease annually [1]. Oral squamous cell carcinoma (OSCC) is a subtype of HNSCC that ranks among the most prevalent malignancies of the head and neck region. OSCC predominantly occurs in Asia and its prevalence is higher in males than females [2]. The current cornerstone therapeutic interventions for OSCC are surgical resection, chemotherapy, and radiotherapy. However, these interventions—and especially combined chemotherapy and radiotherapy—are associated with poor prognoses and therapeutic efficacies, due to approximately 60% of OSCC cases being diagnosed at advanced stages (III and IV), often with regional lymph node involvement. Consequently, the 5-year survival rate for advanced-stage OSCC patients remains as low as 30%. Early-stage diagnosis dramatically improves patient outcomes, with survival rates nearing 90% following appropriate multimodal treatment [3]. Thus, there is an urgent clinical need to identify reliable biomarkers for facilitating early detection and hence improving the prognosis of OSCC [4,5].

The main risk factors for OSCC include tobacco use, excessive alcohol consumption, and betel quid chewing, all of which contribute to the carcinogenesis of oral tissues [6]. Additionally, human papillomavirus (HPV) infection, particularly with high-risk subtypes such as HPV16/18, has been implicated in a subset of OSCC cases, further emphasizing the diverse etiological factors contributing to OSCC malignancy [7]. Betel quid chewing is particularly prevalent in Taiwan [8]. These risk behaviors support the accumulation of genetic mutations and epigenetic alterations, thereby promoting the progression from oral precancerous lesions to invasive malignancies.

Despite the extensive understanding of these risk factors, the exact mechanisms underlying carcinogenesis—particularly the interactions among diverse molecular pathways—remain unclear. A thorough understanding of the genetic alterations implicated in the pathogenesis of oral cancer is crucial, which might not only clarify the underlying mechanisms of tumorigenesis but also lead to the identification of biomarkers and the development of more-effective therapeutic strategies, ultimately improving patient outcomes.

Recent research has provided strong evidence for the involvement of epigenetic regulation in the onset and progression of cancer [9,10,11]. Abnormal DNA hypermethylation in the promoter region of tumor suppressor genes and dysregulated microRNA (miRNA) expression are detected early in the formation of tumor cells, and have been shown to influence carcinogenesis and tumor malignancy [12,13]. We explored epigenetic changes in oral cancer using the Shiny Methylation Analysis Resource Tool (SMART), which provides DNA methylation data for 33 types of cancer in the TCGA database [14]. This tool has revealed that miR-124-3, a member of the miR-124 family, is hypermethylated in tumor tissues relative to their corresponding normal tissues across various cancers, including HNSCC.

miRNAs are small noncoding RNAs involved in RNA silencing and the posttranscriptional regulation of gene expression [15]. In cancer research, miRNAs are considered crucial signaling components in tumorigenesis and appear increasingly promising as disease diagnostic biomarkers [16,17]. Although various miRNAs can function as oncogenes or tumor suppressors in different tumor types, there is substantial evidence that miR-124 acts as a tumor suppressor in several types of cancer, including HNSCC, glioblastoma, gastric cancer, hepatocellular carcinoma, colorectal cancer, and lung cancer. miR-124 is involved in inhibiting the proliferation, migration, and invasion of tumor cells, and even enhancing their chemosensitivity [18,19,20,21,22,23,24]. In OSCC, miR-124 has been shown to suppress cell motility by downregulating the expression of integrin beta-1, a membrane receptor involved in cell adhesion, which reduces the adherence and motility OSCC cells [25]. In addition to its role in regulating OSCC cell motility, miR-124 downregulation has also been observed in an animal model of carcinogen-induced oral cancer, further underscoring its potential involvement in oral carcinogenesis [26]. However, its altered methylation status and functional implications in OSCC have not been fully investigated.

Leucine-rich repeat-containing 1 (LRRC1) is characterized by 14 exons and is responsible for encoding 524 amino acid residues, including 16 leucine-rich repeat sequences and a LAP-specific domain [27]. Similar to other leucine-rich repeat-containing proteins, such as SCRIB, LRRC1 is located at the basolateral side of epithelial cells and involved in the regulation of cell polarity. There is increasing evidence implicating LRRC1 in the progression of certain cancers, including cholangiocarcinoma, hepatocellular carcinoma, and acute myeloid leukemia (AML) [28,29,30]. However, the relationship between miR-124-3 and LRRC1 as well as the function of LRRC1 in OSCC remain unclear.

This study applied the Illumina Infinium MethylationEPIC BeadChip assay to profile 853,307 CpG sites, including >350,000 located in regulatory regions, and other genomic features [31] in 23 paired OSCC and normal tissues. We observed that the methylation level was significantly higher in OSCC tissues than in the normal oral tissues at all 13 CpG sites of miR-124-3 analyzed using the assay. The methylation data were validated by further analyzing the methylation status of miR-124-3 in 18 additional paired OSCC and normal tissues by using a pyrosequencing assay. In addition, we found that the expression level of miR-124-3 was low in OSCC cell lines, and restoring miR-124-3 expression in OSCC cells inhibited the proliferation, colony formation, and migration of these cells, highlighting its potential role in suppressing OSCC tumorigenicity. Applying a luciferase reporter assay identified LRRC1 as a direct target gene of miR-124-3, and lentiviral-vector-mediated knockdown of LRRC1 in OSCC cells decreased their proliferation and migration. These findings reveal the roles and mechanisms of miR-124-3 methylation and its target gene LRRC1 in OSCC, underscoring their potential as biomarkers and therapeutic targets.

## 2. Methods

### 2.1. Sample Collection and Bisulfite Conversion of Genomic DNA

The paired OSCC and adjacent normal tissues used in this study were collected from patients after obtaining informed consent in accordance with a protocol approved by the Institutional Review Board of China Medical University Hospital, Taiwan (IRB no. CMUH102-REC1-054). Genomic DNA of these tissues was extracted using the Gentra Puregene tissue kit (Qiagen, Hilden, Germany) and subjected to bisulfite conversion using the EZ DNA methylation kit (Zymo Research, Irvine, CA, USA). Bisulfite-converted Universal Methylated Human DNA (Zymo Research) was used as an in vitro methylated DNA (IVD) control for the methylation level, determined using the pyrosequencing assay.

### 2.2. Bisulfite Pyrosequencing Assay

Bisulfite-converted DNA was amplified using the following primer pair specific to the miR-124-3 promoter that targeted the region from −191 to +19 relative to the miR-124-3 transcription start site (TSS): 5′-GAAAGGGGAGAAGTGTGGGTTTT-3′ (forward) and 5′-biotin-ACACCCAAAAAAACCCTCAAAACTAAA-3′ (reverse). The amplified PCR products were sequenced using the PyroMark Gold Q24 reagents and the PyroMark Q24 DNA sequencer (Qiagen) with the sequencing primer 5′-GGAGGATTGGGATAGTATA-3′ following the manufacturer’s instructions. The level of miR-124-3 methylation was calculated and then normalized as a percentage using the IVD standard.

### 2.3. Cell Culture

The OSCC cell lines OC2 and OCSL, established from two Taiwanese male patients with a history of betel nut chewing, alcohol consumption, and smoking, were grown in RPMI 1640 medium (Gibco, Grand Island, NY, USA) supplemented with 10% fetal bovine serum (FBS, Invitrogen; Thermo Fisher Scientific, Waltham, MA, USA). The OSCC cell line SCC25 was grown in a 1:1 mixture of DMEM (Gibco) and Ham’s F12 Nutrient Mixture (Gibco) supplemented with 10% FBS, 2.5 mM L-glutamine, 15 mM HEPES, 0.5 mM sodium pyruvate and 400 ng/mL hydrocortisone (Sigma–Aldrich, Burlington, MA, USA). The OSCC cell line HSC3 was grown in a 1:1 mixture of DMEM and Ham’s F12 Nutrient Mixture supplemented with 10% FBS. The human prostate cancer cell line 22Rv1 was maintained in RPMI 1640 medium supplemented with 10% FBS. The transformed human embryonic kidney cell line 293T was maintained in DMEM supplemented with 10% FBS.

### 2.4. RNA Extraction and Quantitative Reverse-Transcription PCR

Total RNA was isolated from cell lines using the REzol C & T reagent (Protech Technology Enterprise, Taipei, Taiwan) following the manufacturer’s instructions. Portions of the extracted RNA were used to synthesize complementary DNA (cDNA) with the Mir-X miRNA first-strand synthesis kit (Clontech, Mountain View, CA, USA). Quantitative reverse-transcription PCR (RT–PCR) was implemented using the ABI StepOne real-time PCR system (Applied Biosystems, Waltham, MA, USA) under the following conditions: initial denaturation at 95 °C for 5 min, followed by 40 cycles of 95 °C for 30 s, 66 °C for 30 s, and 72 °C for 45 s. The following primers were used for miR-124-3 and LRRC1 cDNA amplification: 5′-AGGGCCCCTCTGCGT-3′ (miR-124-3 forward), 5′-GGAGGCGCCTCTCTTGG-3′ (miR-124-3 reverse), 5′-CAGACTAACTCGGATACCTGCAG-3′ (LRRC1 forward), and 5′-CTGGTTGTCAGATAGCCACAGAG-3′ (LRRC1 reverse). GAPDH was also amplified as an internal control using the primers 5′-TTGACGGTGCCATGGAATTT-3′ and 5′-GCCATCAATGACCCCTTCATT-3′. The expression levels of miR-124-3 and LRRC1 were normalized to that of GAPDH.

### 2.5. 5-Aza-2′-Deoxycytidine Treatment

OSCC cells were seeded at a density of 1.5 × 10^5^ in 6-cm culture dishes. After allowing the cells to adhere for 4 h, DMSO, 0.5 μM 5-aza-2′-deoxycytidine (5-Aza-dC), or 2 μM 5-Aza-dC (Sigma–Aldrich) was added. After 72 h of exposure, cells were harvested for DNA, RNA, and protein extraction.

### 2.6. Cell Proliferation Assay

Cells were seeded onto replicate 96-well plates at a density of 2000 cells/well. The cell proliferation was assessed at different time points (4, 24, 48, and 72 h) using the MTS assay with the CellTiter Aqueous One Solution Cell proliferation assay kit (Promega, Madison, WI, USA). The OD (optical density) of cells was determined using a 96-well microplate reader at 492 nm (Revelation, Dynex Technologies, Chantilly, VA, USA).

### 2.7. Colony Formation Assay

Five hundred OSCC cells were seeded in six-well plates and allowed to grow for 1 week or until visible colonies had formed. Cells were then fixed using methanol for 15 min, stained with Giemsa solution (Sigma–Aldrich) for 30 min, and rinsed with ddH_2_O. Colonies were counted by visual inspection under a microscope.

### 2.8. Transwell Cell Migration Assay

A cell migration assay was implemented using 24-well cell culture insert plates (Millicell, Millipore, Burlington, MA, USA). RPMI 1640 medium with 10% FBS was added to the lower chamber of the 24-well plate. In the upper transwell insert, 2 × 10^4^ cells were seeded in RPMI 1640 medium containing 0.5% FBS. OCSL and OC2 cells were allowed to migrate for 16 and 36 h, respectively. The transwell insert was washed with PBS, fixed with 4% formaldehyde for 10 min, and stained with Giemsa solution for 15 min. Following staining, the transwell insert was rinsed with ddH_2_O and the cells on the apical side were removed using a cotton swab. Cell migration was quantified by counting cells under a light microscope with a 100× objective.

### 2.9. Western Blot Assay

Cells were lysed using RIPA Lysis and Extraction Buffer with a protease inhibitor cocktail (Thermo Fisher Scientific) following the manufacturer’s instructions. The protein samples from the lysates were separated by SDS–PAGE and subsequently transferred to PVDF membranes. The membranes were incubated with primary antibodies against LRRC1 (Abcam, Cambridge, UK) and β-actin (Sigma–Aldrich) followed by with secondary antibodies conjugated with horseradish peroxidase, and were then visualized with Amersham Hyperfilm ECL (Cytiva, Marlborough, MA, USA).

### 2.10. Construction of Plasmid for Transient Transfection

The DNA of human miR-124-3 (~0.4 kb) was amplified from 293T cells using the PCR with the primers 5′-TTTGGATCCGAAAGGGGAGAAGTGTG-3′ and 5′-TTTAAGCTTGTTCGCCGGATTTGT-3′. The DNA was then cloned into the pSilencer 4.1-CMV vector (Thermo Fisher Scientific) at the BamHI and HindIII sites to create the pSilencer_miR-124-3 plasmid. After transient transfection with the pSilencer 4.1-CMV vector or the pSilencer_miR-124-3 plasmid using Lipofectamine 2000 (Invitrogen, Carlsbad, CA, USA), OSCC cells were selected by incubating them with 2 μg/mL puromycin (Sigma–Aldrich) for 2 days.

### 2.11. Construction and Production of Lentiviral Vector

To construct a lentiviral vector expressing miR-124-3, the same miR-124-3 DNA fragment was inserted into the pLKO_AS1008 lentiviral vector plasmid (obtained from National RNAi Core Facility, Taiwan), which yielded the pLKO_AS1008_miR-124-3 plasmid. The pLKO_AS1008 or pLKO_AS1008_miR-124-3 plasmid was then co-transfected with lentiviral packaging plasmids pCMVdeltaR8.91 and pMD.G into 293T cells. At 48 h post-transfection, the lentivirus-containing supernatants were collected for use in transduction assays. Successful infection of LKO_AS1008 or LKO_AS1008_miR-124-3 was monitored by GFP expression, and the infected cells were sorted using a cell sorter (FACSAria III, BD Bioscience, Franklin Lakes, NJ, USA).

A lentiviral vector expressing short hairpin RNA (shRNA) was produced by transfecting pCMVdeltaR8.91, pMD.G, and pLKO.1-puro plasmid carrying an shRNA (obtained from National RNAi Core Facility, Taiwan) into 293T cells. The shRNA sequences specific for LRRC1 and GFP were 5′-GAACTAGATGTGTCTCGAAAT-3′ and 5′-CAACAGCCACAACGTCTATAT-3′, respectively. OSCC cells infected with the shLRRC1-expressing lentiviral vector were selected by treatment with 2 μg/mL puromycin (Sigma–Aldrich) for 2 days. Cells infected with the shGFP-expressing lentiviral vector were used as a control.

### 2.12. Construction of Luciferase Reporter Plasmids and Luciferase Activity Assay

The pmirGLO plasmid (Promega) was used to construct luciferase reporter plasmids containing the wild-type or mutated LRRC1 3′ untranslated region (3′UTR). The wild-type LRRC1 3′UTR fragment and its mutated versions were cloned into pmirGLO at the SacI and XbaI restriction sites to generate pLRRC1_WT, pLRRC1_site1_mut, and pLRRC1_site2_mut, respectively. The mutated LRRC1 3′UTR fragments were generated using the Q5 Site-Directed Mutagenesis Kit (New England Biolabs, Ipswich, MA, USA) with the following LRRC1 site1 and site2 mutation primers designed using NEBaseChanger: 5′-GAATCTCATCCCGCAACCAGTC-3′ (site1 mutation forward), 5′-CGTGTTCCTGGGAGCAGCAGC-3′ (site1 mutation reverse), 5′-GAATCTGTATCCTGTGTCATGTC-3′ (site2 mutation forward), 5′-CGTGAAGTGGATGGTACAAATAAAAAC-3′ (site2 mutation reverse). The pmirGLO, pLRRC1_WT, pLRRC1_site1_mut, and pLRRC1_site2_mut constructs were transfected into LKO_AS1008- or LKO_AS1008_miR-124-3-transduced OSCC cells. Luciferase activity was quantified at 48 h post-transfection using a dual-luciferase reporter assay (Promega) according to the manufacturer’s protocol.

### 2.13. Statistical Analyses

Methylation levels were compared between paired normal and tumor groups using paired *t*-tests. The *t*-tests were used to compare methylation levels, gene expression, and the proliferation, colony formation, and migration of cells between two independent groups. Receiver operating characteristic (ROC) curves and the areas under the ROC curves (AUCs) were calculated to quantify the accuracy of using the methylation level for detecting OSCC. Differences at *p* < 0.05 were deemed significant. All analyses were performed using SAS (version 9.4, SAS Institute), and data were visualized using the Matplotlib library (version 3.9.2) in Python (version 3.12.6).

## 3. Results

### 3.1. miR-124-3 Is Frequently Hypermethylated in OSCC Tissues

**Figure 1:**
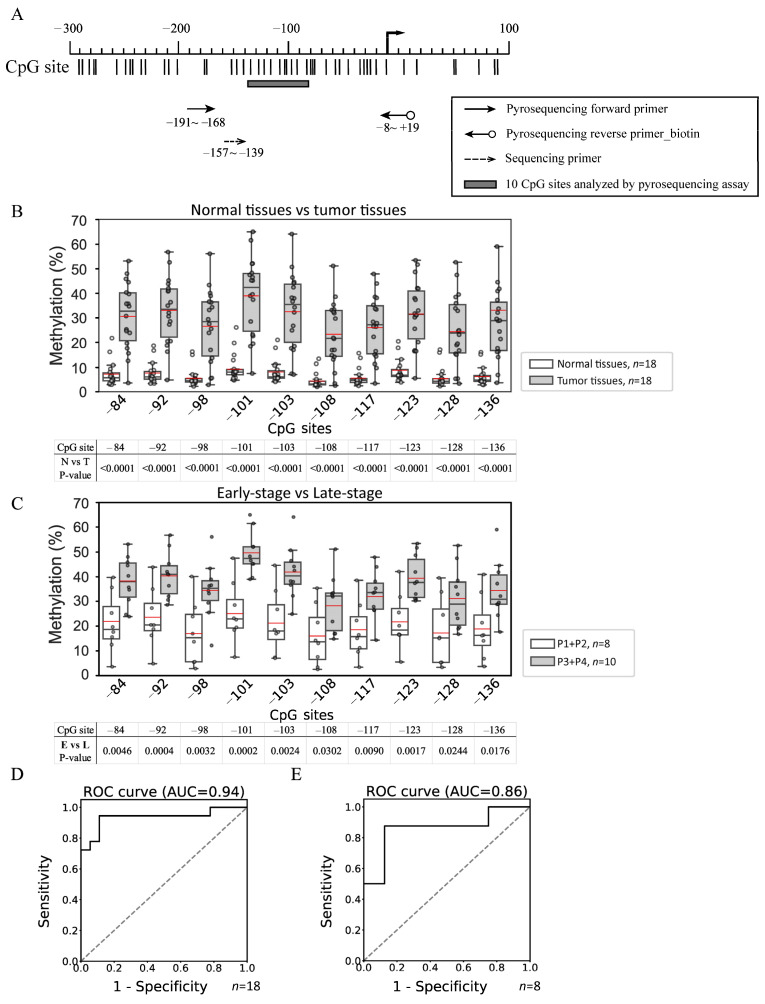
Pyrosequencing analysis of the methylation status in the promoter regions of miR-124-3. (**A**) The region from −191 to +19 was amplified using PCRs, and the area between −80 and −138, which covers 10 CpG sites, was sequenced using a pyrosequencing primer. The distribution of CpG sites is indicated by vertical bars. (**B**) The methylation status of miR-124-3 was analyzed in 18 paired OSCC and adjacent normal tissue samples. In each box plot, the whiskers represent the 10th and 90th percentiles, the box indicates the median with the 25th and 75th percentiles, and the red line is the mean. (**C**) Methylation levels were further compared between early-stage and late-stage tumor tissues. (**D**) The ROC curve obtained using miR-124-3 methylation to detect all-stage OSCC showed an AUC of 0.94. (**E**) The ROC curve obtained using miR-124-3 methylation to detect early-stage (P1 + P2) OSCC showed an AUC of 0.86. N vs. T, normal tissue vs. tumor tissue. E vs. L, early-stage tumor tissue vs. late-stage tumor tissue.

Methylation changes in OSCC tissues were identified precisely by applying the Infinium MethylationEPIC BeadChip assay to 23 paired OSCC and adjacent normal tissue specimens. The characteristics of the included patients are summarized in Table 1A. All 13 CpG sites of miR-124-3 analyzed using the assay exhibited significantly higher methylation levels in OSCC tissues than in normal oral tissues (*p* < 0.0001, Table 2). The methylation data were validated by subjecting an additional set of 18 paired OSCC and adjacent normal tissue samples (Table 1B) to pyrosequencing analysis of the sequence upstream of the miR-124-3 TSS from −80 to −138, which covers 10 CpG sites (Figure 1A). The methylation levels at all 10 CpG sites assayed were significantly higher in tumor tissues than in their counterpart normal tissues (*p* < 0.0001, Figure 1B). In addition to the observed differences in methylation levels between normal and tumor tissues, the miR-124-3 methylation level was higher in late-stage (P3 + P4) than early-stage (P1 + P2) tumor tissues across all CpG sites (Figure 1C), indicating a positive correlation between increased miR-124-3 methylation and progression of OSCC malignancy.

The methylation status of miR-124-3 determined using the pyrosequencing assay appeared to be a promising candidate biomarker for detecting OSCC. The AUC for using methylation levels to detect all-stage OSCC was 0.94 (Figure 1D) and, to detect early-stage OSCC, 0.86 (Figure 1E). These results suggest that the hypermethylation of miR-124-3, as observed in early-stage tumor tissues, could serve as a valuable biomarker for the early detection of OSCC.

### 3.2. Regulation of miR-124-3 Expression by DNA Methylation

**Figure 2:**
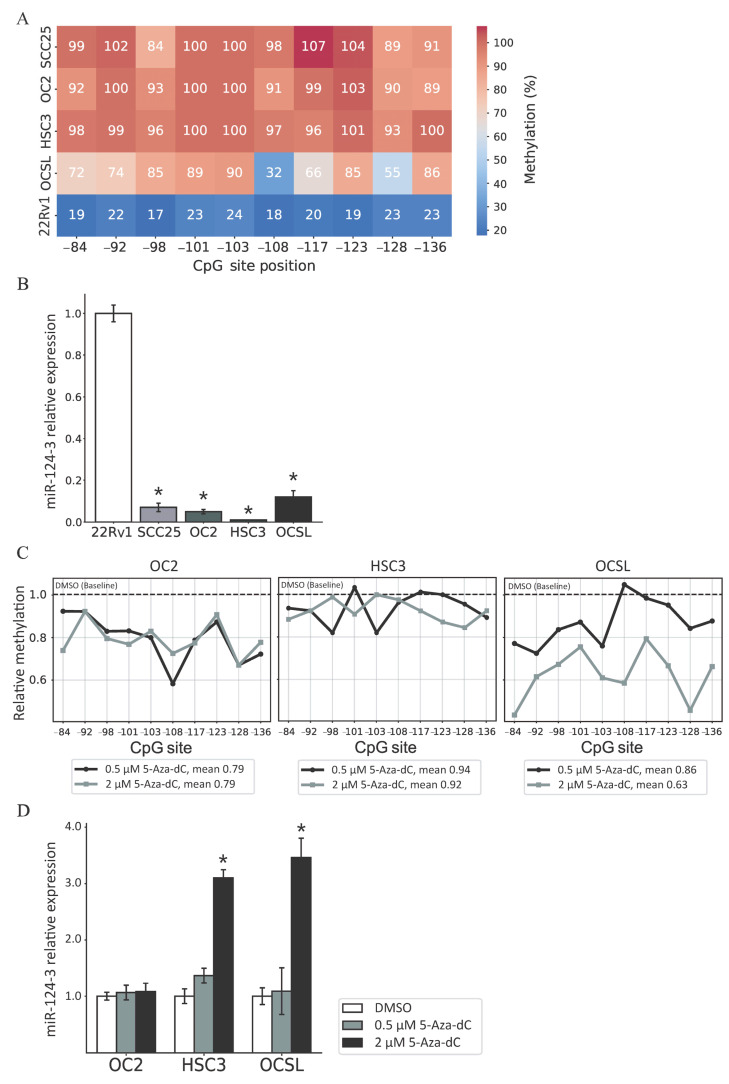
Regulation of miR-124-3 expression by DNA methylation. (**A**) Pyrosequencing assay of the miR-124-3 methylation status in the OSCC cell lines and the 22Rv1 cell line. The methylation level of each of the 10 CpG sites was normalized as a percentage using the IVD standard (given as 100%). (**B**) Relative expression levels of miR-124-3 in the OSCC cell lines. The expression level of miR-124-3 in OSCC cells was normalized to that of the 22Rv1 cell line. (**C**) The methylation levels of the 10 CpG sites in miR-124-3 were measured using a pyrosequencing assay after 5-Aza-dC treatment. Cells were treated with DMSO, 0.5 μM 5-Aza-dC, or 2 μM 5-Aza-dC for 3 days. After normalization using IVD standard, the methylation level of each of the 10 CpG sites was expressed relative to that in the DMSO-treated group. (**D**) The expression level of miR-124-3 in the OSCC cell lines after 5-Aza-dC treatment was measured using a quantitative RT–PCR. The expression level of miR-124-3 in the OSCC cell lines was compared with that of DMSO-treated cells. Data are mean and standard deviation values. * *p* < 0.05.

A pyrosequencing assay was also used to analyze the methylation levels of miR-124-3 promoter in the OSCC cell lines SCC25, OC2, HSC3, and OCSL. It is known that the 22Rv1 cell line exhibits low methylation levels in miR-124-3, and so 22Rv1 was used as the control cell type in the pyrosequencing assay [32]. The results showed that the normalized methylation levels of CpG sites were higher in the four OSCC cell lines than in the 22Rv1 cell line (Figure 2A). A high methylation level in the DNA promoter region is one of the key contributors to gene silencing, and so we performed quantitative RT–PCRs to measure the expression levels of miR-124-3 in these OSCC cell lines. The expression level of miR-124-3 was significantly lower in all four OSCC cell lines than in 22Rv1 (Figure 2B).

We then treated the OSCC cell lines with the demethylating agent 5-Aza-dC to evaluate whether reducing the promoter methylation levels could restore miR-124-3 expression. The pyrosequencing assay demonstrated a reduction in methylation levels across 10 CpG sites in the miR-124-3 promoter region following 5-Aza-dC treatment (Figure 2C). In OC2 cells, the mean methylation level across the 10 CpG sites decreased from 1 in the DMSO control group to 0.79 and 0.79 following treatment with 0.5 and 2 μM 5-Aza-dC, respectively; the corresponding changes were to 0.94 and 0.92, respectively, in HSC3 cells, and to 0.86 and 0.63 in OCSL cells. Although treatment with 5-Aza-dC could reduce the methylation level of miR-124-3 in these OSCC cell lines, the changes in miR-124-3 expression in HSC3 and OCSL cells were only significant for the higher concentration of 5-Aza-dC, and that in OC2 cells was not significant at either concentration (Figure 2D).

### 3.3. MiR-124-3 Inhibits the Proliferation and Migration of OSCC Cells

**Figure 3:**
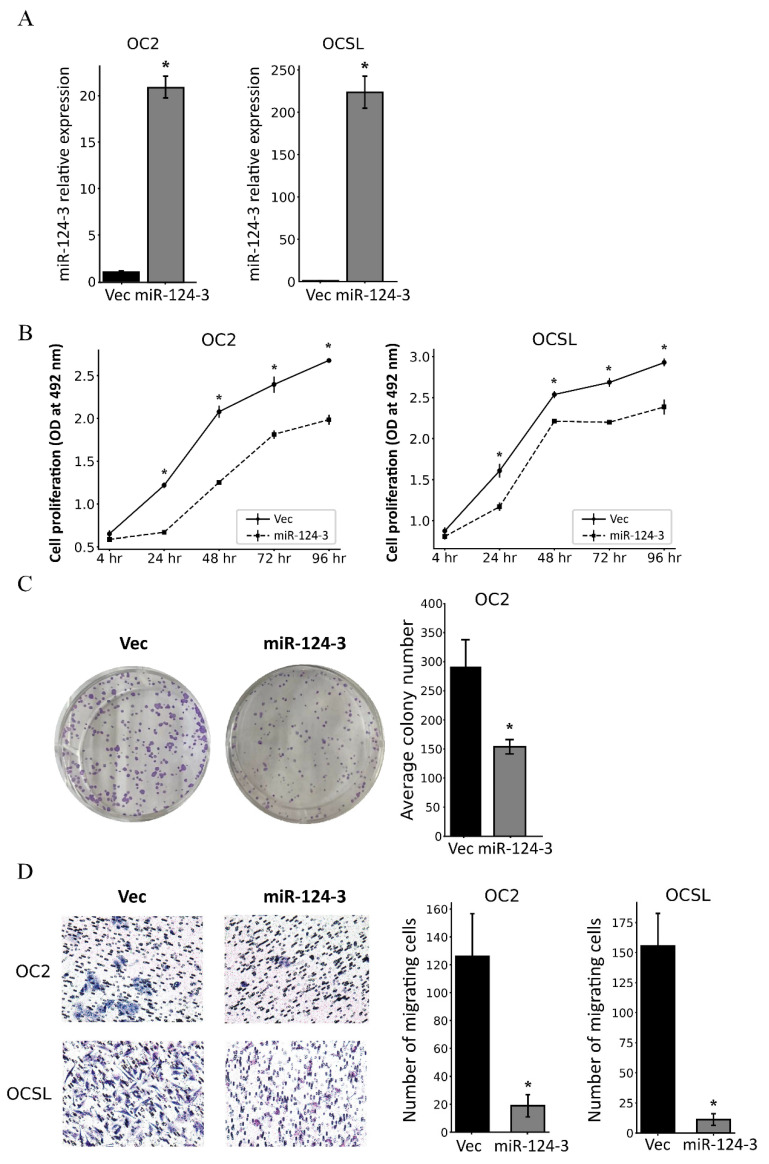
Restored miR-124-3 expression suppressed the proliferation, colony formation, and migration of OSCC cells. (**A**) Graph showing the results of quantitative RT–PCR analyses of miR-124-3 RNA levels in cells transfected with pSilencer 4.1-CMV vector or pSilencer_miR-124-3, with pSilencer 4.1-CMV vector-transfected cells set to 1. (**B**) The effect of miR-124-3 on the proliferation of OSCC cells was assessed using the MTS assay. OD values of cells were read using the Revelation 96-well microplate reader at 492 nm. (**C**) The cell colony formation assay showed that OC2 cells transfected with pSilencer_miR-124-3 formed fewer and smaller colonies. (**D**) The effect of miR-124-3 on OSCC cell migration was evaluated using the transwell migration assay. Data are mean and standard deviation values. * *p* < 0.05. Vec, pSilencer 4.1-CMV vector; miR-124-3, pSilencer_miR-124-3.

To assess the impact of miR-124-3 for OSCC cells, miR-124-3 expression vectors were transfected into OSCC cells and quantitative RT–PCRs were employed to confirm the re-expression of miR-124-3 in the cells (Figure 3A). The MTS assay revealed that overexpression of miR-124-3 significantly inhibited cell proliferation at all time points after 24 h in OC2 and OCSL cells (Figure 3B). The cell colony formation assay also showed that overexpression of miR-124-3 in OC2 cells resulted in significant reductions in both the number and size of the colonies formed (Figure 3C). A cell migration assay was additionally conducted to determine the effect of miR-124-3 on OSCC cell migration, which revealed that miR-124-3 overexpression significantly reduced the migration of OC2 and OCSL cells compared with the control groups (Figure 3D). Together these data indicate that miR-124-3 inhibits the growth and motility of OSCC.

### 3.4. MiR-124-3 Downregulates LRRC1 Expression by Targeting Its 3′UTR

**Figure 4:**
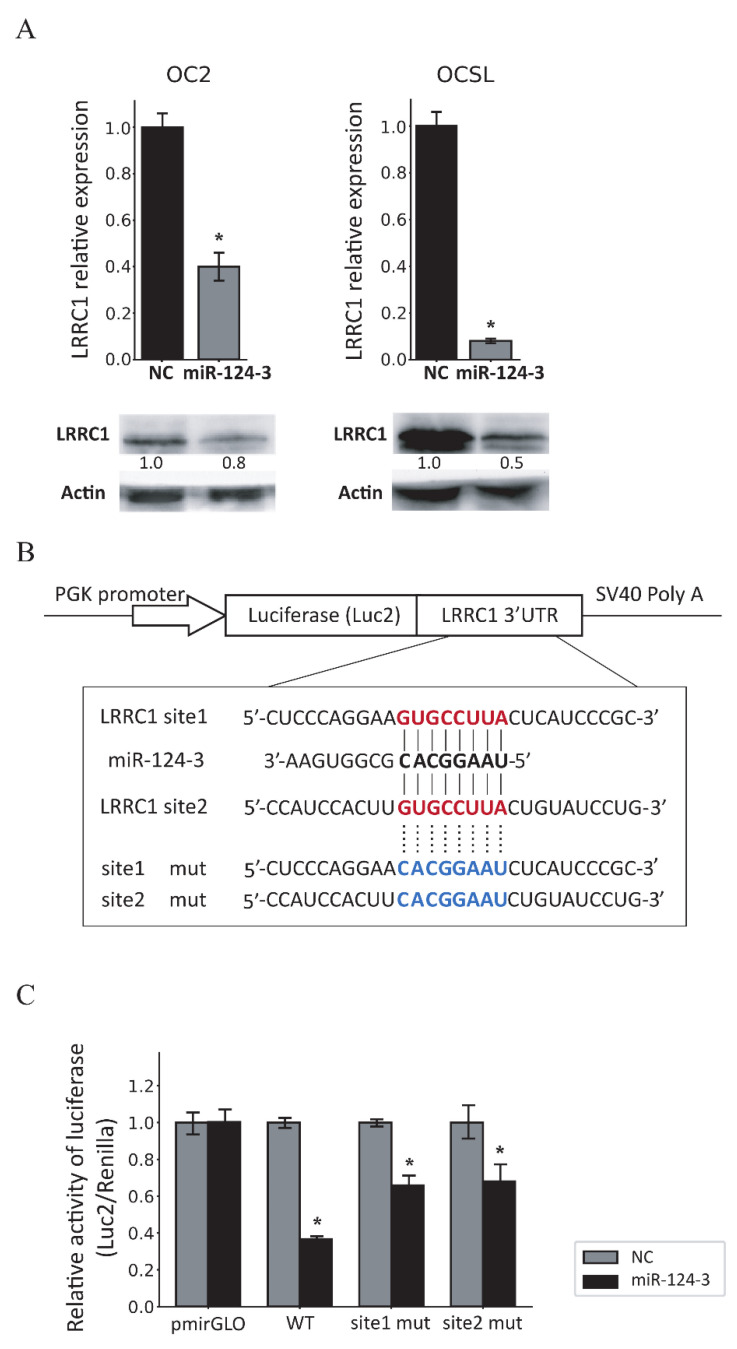
The LRRC1 gene is a direct target of miR-124-3. (**A**) Graph showing the results of quantitative RT–PCR analyses of LRRC1 expression in cells transduced with lentiviral vector LKO_AS1008 or LKO_AS1008_miR-124-3, with LKO_AS1008-transduced cells set to 1. Western blot findings of LRRC1 expression in cells transduced with LKO_AS1008 or LKO_AS1008_miR-124-3, with β-actin used as a loading control. Quantification was performed by normalizing LRRC1 to β-actin and expressing it relative to the NC group. (**B**) Construction of luciferase reporter plasmids containing the wild-type or mutated LRRC1 3′UTR. The sequences of potential miR-124-3 binding sites in the 3′UTR of LRRC1 are shown in red. The corresponding mutated sequences in the LRRC1 3′UTR in pLRRC1_site1_mut and pLRRC1_site2_mut are shown in blue. (**C**) Luciferase activity assay confirmed that LRRC1 was the direct target gene of miR-124-3. The pmirGLO, pLRRC1_WT, pLRRC1_site1_mut, and pLRRC1_site2_mut constructs were transfected into LKO_AS1008- or LKO_AS1008_miR-124-3-transduced OSCC cells. At 48 h post-transfection, luciferase activity was quantified using a dual-luciferase reporter assay. Data are mean and standard deviation values. * *p* < 0.05. NC, LKO_AS1008-transduced cells; miR-124-3, LKO_AS1008_miR-124-3-transduced cells; WT, pLRRC1_WT; site1 mut, pLRRC1_site1_mut; site2 mut, pLRRC1_site2_mut.

We used three databases to identify the target gene through which miR-124-3 inhibits tumor cell growth and migration: TargetScan 7.1, miRDB, and DIANAlab. From each database, we selected the top 50 genes with the highest prediction scores and identified 7 overlapping candidate genes. We then excluded genes with low expression in OSCC, further refined our selection based on literature review, and ultimately focused on LRRC1. It is known that LRRC1 is associated with cancer progression [28,29,30], and its 3′UTR contains two binding sites (GUGCCUUA) for miR-124-3 according to the miRDB database. To confirm whether LRRC1 is a target gene of miR-124-3, we examined the expression of LRRC1 mRNA and protein in miR-124-3-overexpressing cells. The results showed that both LRRC1 mRNA and protein levels were significantly lower in miR-124-3-overexpressing OSCC cell lines than in the control group (Figure 4A; Supplementary Figure S1).

We subsequently used dual-luciferase reporter plasmids containing the wild-type or mutated LRRC1 3′UTR to determine whether miR-124-3 directly binds to the LRRC1 3′UTR to suppress LRRC1 expression (Figure 4B). We found that co-expression of miR-124-3 in OSCC cells significantly suppressed the luciferase activity of the wild-type LRRC1 3′UTR, while a single binding-site mutation of the LRRC1 3′UTR partially rescued the luciferase activity (Figure 4C). These results demonstrate that miR-124-3 inhibits LRRC1 expression by directly binding to the miRNA binding sites within the LRRC1 3′UTR. It was especially notable that both binding sites in that 3′UTR are involved in the regulation of LRRC1 expression.

### 3.5. Knockdown of LRRC1 Inhibits the Proliferation and Migration of OSCC Cells

**Figure 5:**
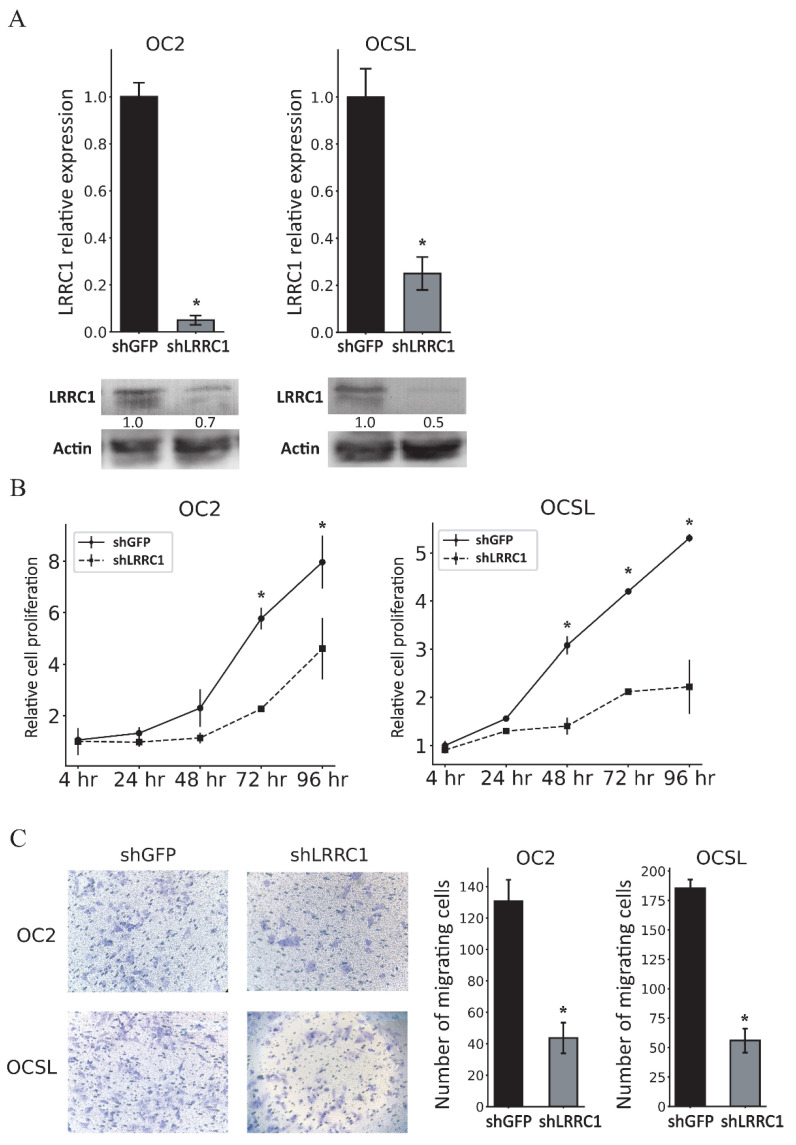
Knockdown of LRRC1 expression significantly reduced the proliferation and migration of OSCC cells. (**A**) Graph showing the results of quantitative RT–PCR analyses performed to assess LRRC1 expression levels in OSCC cells transduced with lentiviral vector carrying shLRRC1 or shGFP. The expression level of each shGFP-transduced cell was assigned a value of 1. Western blot findings of LRRC1 expression in cells transduced with lentiviral vector carrying shLRRC1 or shGFP, with β-actin used as a loading control. Quantification was performed by normalizing LRRC1 to β-actin and expressing it relative to the shGFP group. (**B**) The effect of LRRC1 knockdown on the proliferation of OSCC cells was assessed using the MTS assay. The increases in cell numbers were normalized to the number of cells at 4 h after cell seeding. (**C**) The effect of LRRC1 knockdown on OSCC cell migration was evaluated using the transwell migration assay. Data are mean and standard deviation values. * *p* < 0.05. shLRRC1, shLRRC1-expressing lentiviral vector; shGFP, shGFP-expressing lentiviral vector.

We further investigated the role of LRRC1 in OSCC cell proliferation and migration by using lentiviral-vector-mediated RNA interference to knock down LRRC1 in OSCC cell lines. The expression of LRRC1 was significantly suppressed in terms of the levels of both mRNA and protein after LRRC1 knockdown (Figure 5A; Supplementary Figure S2). Similar to miR-124-3 overexpression, knockdown of LRRC1 in OSCC cell lines reduced both cell proliferation (Figure 5B) and migration (Figure 5C).

## 4. Discussion

DNA methylation holds great promise as a cancer biomarker, having potential not only in detecting early-stage malignancy but also in monitoring disease progression and recurrence posttreatment [33,34,35,36]. The application of SMART to 23 cancer types and their corresponding normal tissues from the TCGA database—of 33 available cancer types in the database, 23 types have been compared with their corresponding normal tissues [14]—identified miR-124-3 with a high methylation level in 17 types of cancer, including HNSCC. In this study we employed a high-throughput methylation microarray to investigate DNA methylation profiles in 23 paired OSCC and normal tissues. Our analyses identified that the methylation level of miR-124-3 (20q13.33) was significantly higher in OSCC tissues than in normal oral tissues. The methylation microarray data also revealed that the two other members of the miR-124 family, miR-124-1 (8p23.1) and miR-124-2 (8q12.3), are highly methylated in OSCC (Supplementary Table S1). Although miR-124 hypermethylation in OSCC has been confirmed in the present study, the sample size remains relatively small for broader applicability of our conclusion. Further studies with large cohorts are necessary to substantiate these findings.

Pyrosequencing analysis revealed that the miR-124-3 methylation level was markedly higher in late-stage than early-stage tumors. We additionally identified a significant difference in methylation level between normal and early-stage tumor tissues, quantified as an AUC of 0.86 for distinguishing early-stage tumors from normal tissues, indicating that miR-124-3 methylation is an early event in OSCC development. This underscores the potential of miR-124-3 methylation profiling as a clinical biomarker for early OSCC detection. All 10 CpG sites analyzed using a pyrosequencing assay showed significant differences in methylation levels between early and late stages, indicating that methylation increased as the oral tumorigenesis progressed.

The role of DNA methylation in suppressing miR-124 expression has been reported in breast, colon, and gastric cancers [37,38,39]. We assessed whether miR-124-3 RNA expression in OSCC is impacted by DNA methylation by treating the OC2, HSC3, and OCSL OSCC cell lines with 5-Aza-dC. This treatment reduced miR-124-3 methylation levels in all three cell lines tested, leading to increased expression of miR-124-3 in HSC3 and OCSL cells but not in OC2 cells. Applying 5-Aza-dC at a higher concentration (>2 μM) might induce a larger reduction of methylation so as to reactivate miR-124-3 expression in OC2 cells. In addition, previous studies have found that combined treatment with 5-Aza-dC and the histone deacetylase inhibitor trichostatin A synergistically induced the re-expression of some epigenetically silenced genes [40,41,42]. However, we found that combined treatment did not significantly reduce miR-124-3 methylation or increase miR-124-3 expression in the OSCC cell lines tested, indicating that DNA methylation is the main mechanism underlying the suppression of miR-124-3 expression in OSCC.

It has been reported that miR-124 is downregulated and acts as a tumor suppressor in various cancer types [43]. Our application of bioinformatics tools to investigate the tumor-suppressive mechanisms of miR-124-3 in OSCC identified LRRC1 as a potential target of miR-124-3. Our luciferase activity assay confirmed that LRRC1 is a direct target of miR-124-3 in OSCC, with miR-124-3 specifically inhibiting LRRC1 expression through binding to its 3′UTR. Furthermore, knockdown of LRRC1 expression led to tumor-suppressive effects similar to those induced by overexpression of miR-124-3 in OSCC. Similarly, LRRC1 has been shown to promote tumor progression in AML, with its knockdown inhibiting proliferation and glycolysis while promoting apoptosis [29]. Moreover, circ-ZNF609 promotes tumor progression in cholangiocarcinoma by upregulating LRRC1 via targeting miR-432-5p, leading to increased proliferation, migration, and invasion [30]. To further investigate the clinical significance of LRRC1, we explored the TCGA database for identification of potential prognostic genes in HNSCC and our analysis reveals that LRRC1 expression was significantly higher in tumor tissues than in normal tissues. However, its association with patients’ prognosis remains unclear, as survival analysis of HNSCC patients did not show a strong correlation between LRRC1 expression level and overall survival.

Despite this, our findings suggest that targeting LRRC1 through miRNA-based approaches such as miR-124-3 may have therapeutic potential. Indeed, therapeutic strategies using miRNAs have been explored in other cancer types; for example, in cisplatin-resistant lung cancer, bone-marrow mesenchymal stem-cell-derived exosomes shuffle miR-193a has been shown to downregulate LRRC1, thus inhibiting the proliferation, colony formation, invasion, and migration of tumor cells, while promoting apoptosis [44]. This suggests that targeting LRRC1 through miRNA modulation could be an effective strategy across different cancer types. However, to determine the feasibility of this therapeutic strategy, in vivo experiments evaluating how miR-124-3 restoration would affect tumor progression need to be conducted in the future. Nonetheless, despite the lack of in vivo evidence, the outcomes of our in vitro functional assays in the present study are consistent with prior studies [43] identifying miR-124-3 as a tumor suppressor, supporting the potential therapeutic value of miR-124-3 for OSCC.

## 5. Conclusions

The present study concludes that miR-124-3 methylation is not only a potential diagnostic biomarker for early-stage OSCC but also acts as a tumor suppressor by directly targeting LRRC1, thereby inhibiting the proliferation and migration of OSCC cells. Further explorations of additional target genes of miR-124-3 in OSCC may yield promising strategies for cancer therapy.

## Figures and Tables

**Table 1:** Selected patients’ characteristics.

**(A) For Infinium MethylationEPIC BeadChip Assay**		
	Patients	
Characteristics	(*n* = 23)	
Age (years, mean ± SD)	50.6 ± 7.6	
Pathological Stage	*n*	%
P1	3	13.0%
P2	5	21.8%
P3	8	34.8%
P4	7	30.4%
**(B) For Pyrosequencing Assay**		
	Patients	
Characteristics	(*n* = 18)	
Age (years, mean ± SD)	54.5 ± 10.9	
Pathological Stage	*n*	%
P1	3	16.7%
P2	5	27.8%
P3	6	33.3%
P4	4	22.2%

SD: standard deviation.

**Table 2:** Performance of 13 CpG sites of miR-124-3 for detecting OSCC.

Probe	UCSC RefGene Group	Mean Δβ	Discrimination Statistics			
			AUC	(95% CI)		*p*
cg08737296	TSS1500	0.14	0.91	(0.82,	0.99)	<0.0001
cg02650317	TSS1500	0.22	0.88	(0.77,	0.99)	<0.0001
cg02065637	TSS1500	0.30	0.94	(0.87,	1.00)	<0.0001
cg04927004	TSS1500	0.11	0.85	(0.72,	0.97)	<0.0001
cg15699267	TSS1500	0.26	0.90	(0.80,	0.99)	<0.0001
cg20277905	TSS200	0.16	0.89	(0.80,	0.98)	<0.0001
cg19267861	TSS200	0.25	0.91	(0.82,	1.00)	<0.0001
cg03387135	TSS200	0.18	0.85	(0.73,	0.97)	<0.0001
cg01052879	TSS200	0.16	0.89	(0.79,	0.99)	<0.0001
cg18627360	TSS200	0.13	0.90	(0.81,	0.99)	<0.0001
cg15028514	TSS200	0.15	0.90	(0.80,	0.99)	<0.0001
cg06660530	Body	0.16	0.87	(0.77,	0.97)	<0.0001
cg18772588	Body	0.20	0.86	(0.75,	0.97)	<0.0001

Δβ = β_tumor_ − β_normal_; AUC = area under the ROC curve; CI = confidence interval.

## Data Availability

The data generated in the present study may be requested from the corresponding author.

## Supplementary Materials

The following supporting information can be downloaded at: https://www.mdpi.com/article/10.3390/cancers17071136/s1, Figure S1: Western blot findings of LRRC1 expression in cells transduced with LKO_AS1008 or LKO_AS1008_miR-124-3; Figure S2: Western blot findings of LRRC1 expression in cells transduced with lentiviral vector carrying shLRRC1 or shGFP; Table S1: Performance of CpG sites of miR-124-1 and miR-124-2 for detecting OSCC.
