# Supplementary material for: Methylation-Mediated Silencing of miR-124-3 Regulates LRRC1 Expression and Promotes Oral Cancer Progression
Source: Cancers (Basel). 2025 Mar 28;17(7):1136. doi: 10.3390/cancers17071136 (PMC11988110; doi:10.3390/cancers17071136)
Supplement: Supplementary file 1 [file cancers-17-01136-s001.zip › miR124 2025.2.14 Table S1.pdf]

Table S1. Performance of CpG sites of miR-124-1 and miR-124-2 for detecting OSCC.

miR-124-1						
probe	UCSC RefGene Group	Mean Δβ	Discrimination statistics			
			AUC	(95% CI)		*P*
cg00571033	Body	0.12	0.89	0.78	0.99	<.0001
cg01275681	TSS200	0.15	0.78	0.64	0.92	<.0001
cg14278808	TSS200	0.14	0.81	0.68	0.94	<.0001
cg15248835	TSS200	0.15	0.84	0.71	0.96	<.0001
cg12616174	TSS1500	0.16	0.85	0.73	0.97	<.0001
cg06292304	TSS1500	0.05	0.71	0.56	0.86	0.0060
cg24185864	TSS1500	0.11	0.84	0.73	0.96	<.0001
cg18246262	TSS1500	0.05	0.75	0.60	0.91	0.0010
cg15537082	TSS1500	0.00	0.51	0.33	0.69	0.9243

miR-124-2						
probe	UCSC RefGene Group	Mean Δβ	Discrimination statistics			
			AUC	(95% CI)		*P*
cg10698928	TSS1500	0.23	0.89	0.79	0.99	<.0001
cg07792478	TSS1500	0.23	0.85	0.74	0.97	<.0001
cg14590098	TSS1500	0.14	0.86	0.74	0.97	<.0001
cg02226645	TSS1500	0.12	0.80	0.66	0.93	<.0001
cg04104463	TSS1500	0.08	0.75	0.61	0.89	0.0005
cg25900085	TSS200	0.19	0.83	0.71	0.96	<.0001
cg05474726	TSS200	0.17	0.83	0.71	0.96	<.0001
cg27313642	TSS200	0.14	0.88	0.78	0.98	<.0001
cg16189671	TSS200	0.07	0.79	0.65	0.93	<.0001
cg20653075	TSS200	0.14	0.85	0.73	0.97	<.0001
cg05455720	TSS200	0.16	0.85	0.73	0.97	<.0001
cg04559779	Body	0.20	0.84	0.72	0.96	<.0001

$\Delta\beta=\beta_{tumor}-\beta_{normal}$

AUC=area under the ROC curve; CI=confidence interval
