# Supplementary figures and images for: Methylation-Mediated Silencing of miR-124-3 Regulates LRRC1 Expression and Promotes Oral Cancer Progression
Source: Cancers (Basel). 2025 Mar 28;17(7):1136. doi: 10.3390/cancers17071136 (PMC11988110; doi:10.3390/cancers17071136)

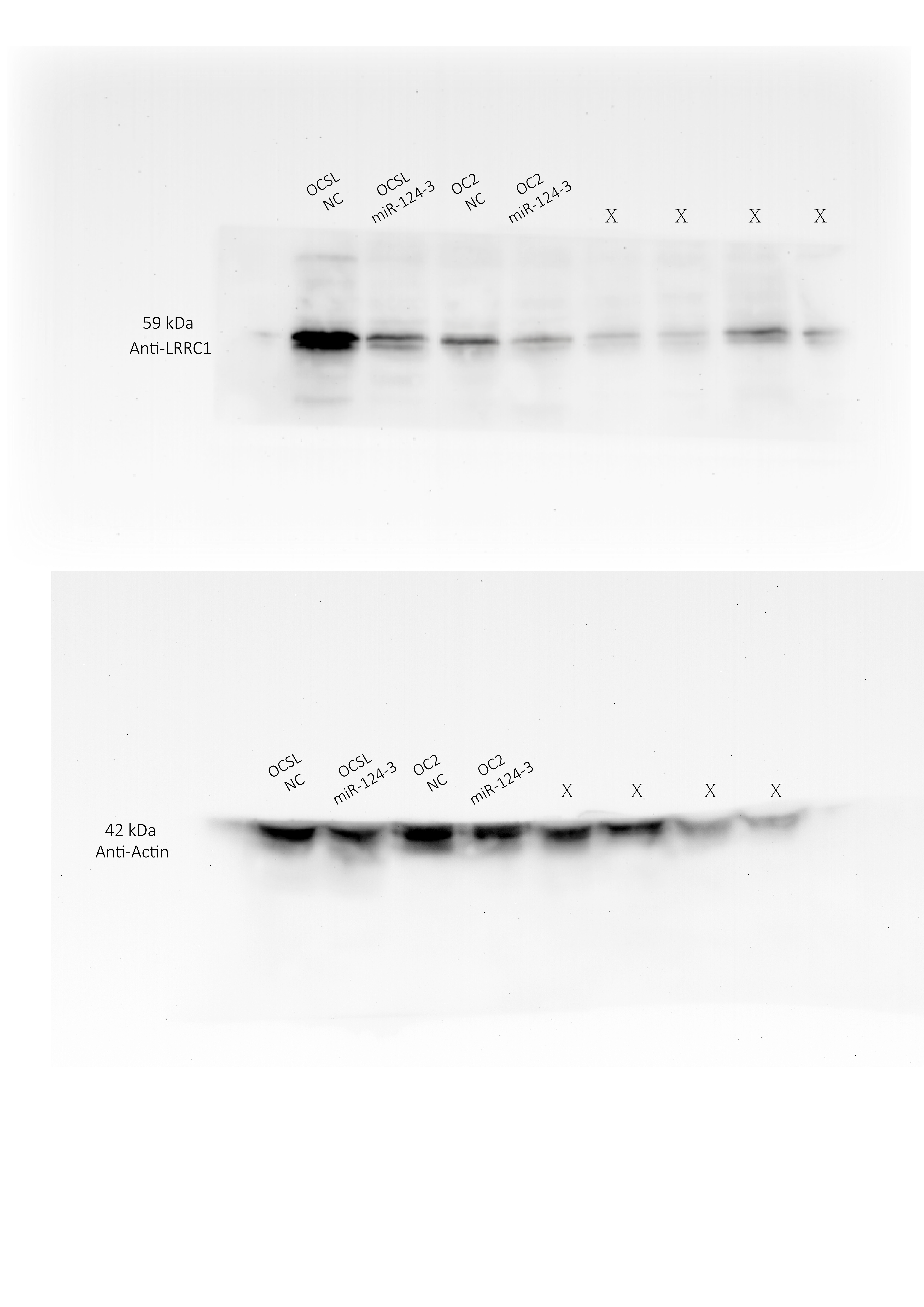

Supplement: Supplementary file 1 [file cancers-17-01136-s001.zip › Figure S1.tif]

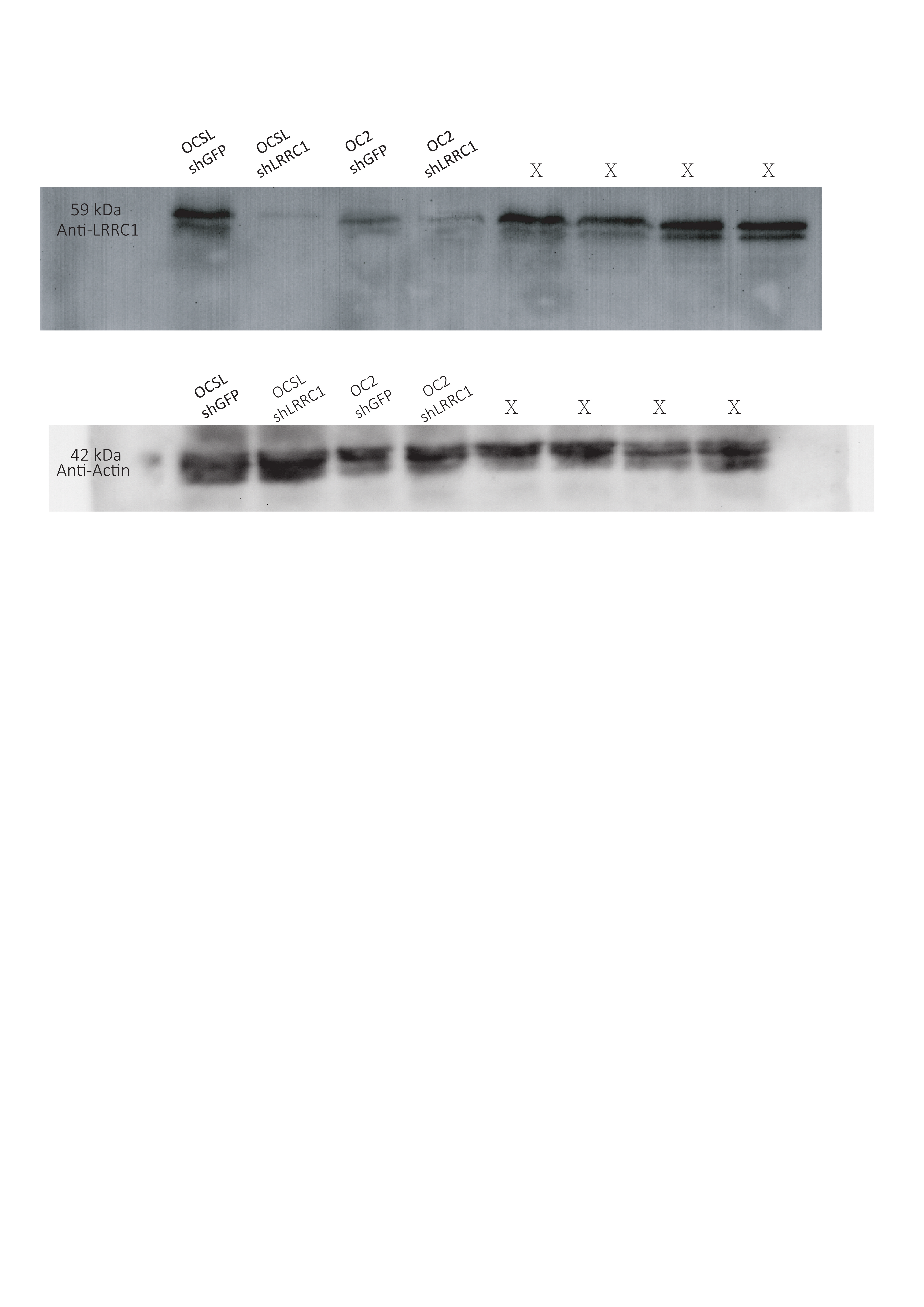

Supplement: Supplementary file 1 [file cancers-17-01136-s001.zip › Figure S2.tif]
